# An In-Depth Analysis of Providers and Services of Cancellation in Anesthesia Reveals a Complex Picture after Systemic Analysis

**DOI:** 10.3390/healthcare11030357

**Published:** 2023-01-26

**Authors:** Krzysztof Laudanski, Justin Wain, Mark-Alan Pizzini

**Affiliations:** 1 Department of Anesthesiology and Perioperative Care, Mayo Clinic, Rochester, MN 55905, USA; 2 School of Osteopathic Medicine, Campbell University, Lillington, NC 27546, USA; 3 Department of Anesthesiology and Critical Care, Hospital of the University of Pennsylvania, Philadelphia, PA 19104, USA

**Keywords:** peri-operative care, workflow, hospital, holidays, cancellation, precision medicine, efficiency, safety, operating room, surgery

## Abstract

The variances in operating room (OR) cancellation rates between different service lines and operators within these service lines were assessed by reviewing the electronic medical record (EMR) covering 34,561 cases performed by 199 OR operators in 2018. We assumed that cancellations would differ between different service lines, but the between-operators variance was minimal within the service line. We hypothesized that most variability would be secondary to patient-specific (weekdays, time of year, and national holidays), seasonal and administrative issues. Of 4165 case cancellations, the majority (73.1%) occurred before the patient arrived at the hospital. A total of 60% of all cancellations were within gastroenterology, interventional cardiology, and orthopedics. Cancellation rate variability between surgeons operating within the same service line greatly varied between services from very homogenous to very diverse across providers. The top reasons for cancellation were: date change, canceled by a patient, or “no show”. The highest cancellation rates occurred on Mondays and Tuesdays, in January and September, and during weeks associated with national holidays. In summary, cancellation variability must be analyzed at the level of individual specialties, operators, and time variability.

## 1. Introduction

Efficient workflow in the operating room (OR) is critical to patient satisfaction, reduction of iatrogenic risk, and fiscal prudence [1,2,3]. One of the most disruptive events is an unexpected case cancellation, with a reported rate of between 0.34% to 44.2%, but diverse methodologies hamper comparisons between studies [2,4,5,6,7,8,9,10,11,12,13,14,15,16,17,18,19,20,21,22,23,24,25,26,27,28]. In most cases, cancellations occur within 24 h of surgery, but they also occur after induction of anesthesia [29]. Rescheduling cases may not necessarily lead to in-creased workload variability, but it does affect patient satisfaction and enterprise bottom line [30]. On the other hand, cancellations may be necessary due to medical reasons, but the provider may face production pressure, leading to unnecessary risks [31,32,33].

Most reports have focused on a particular service line or hospital as a whole [5,8,9,10,11,21,26,27,28,34,35,36,37,38,39,40,41]. However, the performance of individual operators within the service line is primarily unknown. Cancellations have a complex and multimodal nature [2,4,5,8,9,10,11,21,26,27,28,29,31,32,33,34,35,36,37,38,39]. First, patients may not show up for scheduled cases for various reasons, including financial barriers or lack of support [5,13,14,34,35,37,38]. Surgical staff, the procedural room, or supplies may not be available [4,5,11,12,13,14,16,18,19,23,37,40,41]. Third, patient medical problems may lead to cancellations [4,11,13,16,18,26,27,28,31,32,34,35,36,37,38,42]. Finally, administrative causes related to scheduling, clerical errors, insurance authorization, or payment issues are frequently present [2,12,27,29,38,43]. High heterogeneity of the underlying causes in the reported literature is due to regional specifics of healthcare delivery [2,17,20,22,24,28]. However, it is unknown how these reasons vary between different service lines and surgeons within the same service line, representing a knowledge gap. There is also a perception of cancellations being more frequent on certain days or during the holidays. So far, most data have been derived using manual reviews of cancellations and retrospective data, potentially introducing bias [14,32,33]. A large quantity of data obtained from electronic medical records (EMR) may reveal a pattern of cancellation to delineate if the service line, or specific OR operators, should be targeted in remedial stages as the dominant cause of excessive cancellation [30,44,45,46,47,48,49,50,51,52,53,54,55]. Utilizing EMR records entered independently from providers and researchers allows for studying the gap in knowledge in service- vs. operator- vs. seasonal-related cancellation variations more accurately by minimizing observer bias. Ultimately, investigating these issues will help to address specific solutions to reduce the rate of cancellations at the appropriate level of the organizational structure of the healthcare system [1,42].

## 2. Materials and Methods

Electronic medical records (EMR) were used to extract data between January 2018 and December 2018 in a large, urban, academic center involving 199 operating room operators. As this was a quality and implementation project (Q&I), IRB approval and consent were not required. Data analysis was carried out using a standardized workflow (Supplementary Figure S1).

Cancellation records were entered by staff in the administrative, pre-operative, and operative areas in the EPIC™ system (Epic systems, Verona, WI, USA) using pre-determined fields without manual abstraction by the authors of this study. Staff obtained a standard orientation on how to enter the data per hospital policy. The cancellations were divided as: pre-op (cancellation before a patient arrived at the hospital), pre-operative area (patient arrived at the hospital, but he/she was not transferred to the operating room), and OR cancellation (cancellation after a patient reached the OR). Only cancellations before pre-op were allowed to have EPIC-defined explanations entered into the database. Table 1 provides the definitions and descriptive analysis of the different cancellation reasons.

We stratified data by service line and surgical provider. We excluded surgeons with an annual case volume of <10. The U.S. holiday calendar was used for some visualizations.

Statistical analysis was conducted by visualizing measures of average and variable dispersion. The Shapiro-Wilk W test and distribution plots were used to test the normality of distribution variables. The homogeneity of variance was evaluated with Levene′s test. Parametric variables were expressed as mean ±SD and compared using Student’s *t*-test. For non-parametric variables, median (M_e_) and interquartile ranges (I.R.) were shown with the U-Mann-Whitney statistic. Outliers were identified using Grube′s test. A double-sided *p*-value less than 0.05 was considered statistically significant for all tests. Statistical analyses were performed with Statistica 11.0 (StatSoft Inc., Tulsa, OK, USA).

## 3. Results

### 3.1. Overall Cancellation Rates

The total number of cancellations before reaching pre-op was 3045 (73.1% of the total 4165 cancellation cases). A total of 636 (15.3%) cases were canceled after patients reached the pre-operative area, while 33 (0.8%) and 35 (0.8%) were canceled in the operating theater before and after induction, respectively. Cancellation reasons were grouped into three main clusters by batching EPIC entries after removing the cases characterized as duplicates (*n* = 416; 10%) (Table 1). The dominant reasons across all cancellations before the patient reached the pre-op were patient-related (18.4% of total cases) and administrative (19% of total cases), with clinical-related reasons in the minority (14.9%).

### 3.2. Analysis of Cancellation per Surgeon

**Figure 1:**
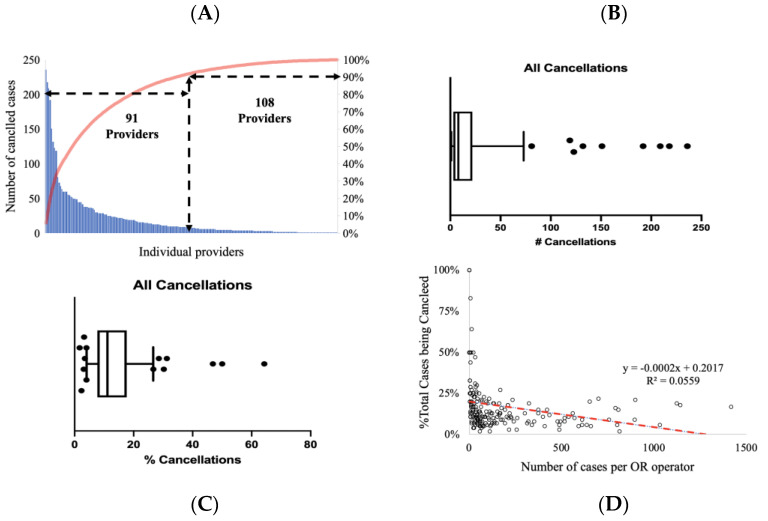
A total of 91 out of 199 providers compromised 90% of all cancellations (**A**), but 9 OR operators had disproportionally high numbers (#) of cancellations per volume (**B**) or percentage (%) of all cancellations (**C**). The volume correlated negatively to % of cancellations (**D**).

We analyzed 34,561 scheduled cases with 4165 cancellations (12%). The average median of canceled cases across all surgeons was eight cases/per year. The ratio of canceled cases over total scheduled cases averaged 12.3% ± 5.99% across all OR operators. A total of 91 providers contributed to 90% of canceled cases (Figure 1A). Even more prominently, four surgeons contributed 20.5% of all cancellations (Figure 1A,C). Their cancellations were not clustered around distinctive periods (data not shown). However, when the data were presented as the % of all cases, we identified two clusters—OR operators under- and over-canceling cases compared to the cohort average (Figure 1B). Distributions analysis demonstrated heterogeneity of anesthesia location operators, with some experiencing a disproportional percentage of cases being canceled or having very low cancellation rates (Figure 1C). A number of canceled cases correlated with provider volume (r = 0.86; *p* = 0.00001) (data not shown). However, the percentage of canceled cases correlated weakly, yet statistically significantly with the total number of cases when all specialties were analyzed together (Figure 1D). However, these correlations somewhat depended on specialty (Supplemental Figure S3).

### 3.3. Analysis of Surgical Cancellation per Service Line

**Figure 2:**
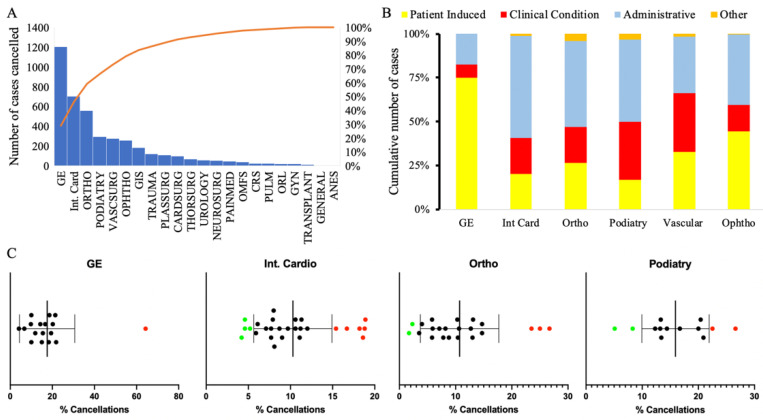
Top six services experienced 90% of cancellation event (**A**) with very high variability in terms of causes (**B**). There was significant variability between surgeons even within a service line (**C**). Abbreviations: gastroenterology (GE), ophthalmology (Ophtho), interventional cardiology (Int. Card). Green dots represent providers who were below the 95% CI and red dots represent providers who were above the 95% CI.

Gastroenterology (GE), interventional cardiology, and orthopedics comprised 59% of all cancellations (Figure 2A). The main reasons for cancellations in these groups were highly variable between the services (Figure 2B; Supplemental Figure S1; Supplemental Table S1).

There was significant variability between different surgeons within each of these services regarding the number of cases canceled. We discovered services with surgeons canceling a disproportionally high and low number of cases in outlier analysis, but these analyses can only be conducted to service lines with more than four operators (Figure 2C).

**Figure 3:**
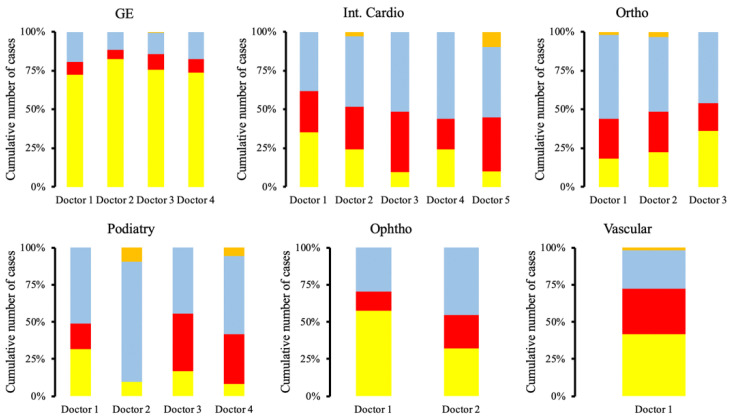
Cancellation reasons are highly variable between operators of the same service. Abbreviations: gastroenterology (GE), ophthalmology (Ophtho), interventional cardiology (Int. Card). Yellow represents cancellations that were patient related; red represents cancellations that were clinical in nature; blue represents cancellations that were secondary to administration; orange represents cancellations that were miscellaneous and did not fit in the other groupings.

Finally, we looked at the practice patterns in cancellations of the outlier operators in GE endoscopy, ophthalmology, interventional cardiology, orthopedics, and podiatry, demonstrating significant variability (Figure 3).

### 3.4. Analysis of Surgical Cancellation per Time and Place

**Figure 4:**
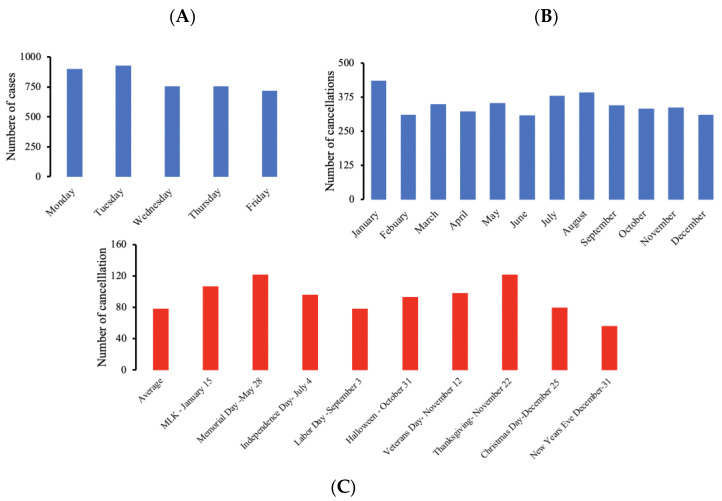
There was a spike in cancellations on Monday and Tuesday (**A**), January and August (**B**), and during certain U.S. Holidays (**C**).

Most of the cancellations occurred on Mondays and Tuesdays, as well as in January and August, but the variation was not statistically significant (Figure 4A,B). Furthermore, cancellation frequency was higher in the week before most holidays, except Labor Day and New Year (Figure 4C). Patients choosing to cancel the surgery or wanting to reschedule the surgery consisted of the majority of cancellations seen on Mondays and Tuesdays, and during the months of January and August. The month of January did have an increase in cancellations due to patients not showing up for the surgery, while August had an increase in cancellations in the pre-operative setting. Finally, the cancellation per surgeon over time followed a somewhat individualized pattern, but evened out over time (Supplementary Figure S4).

## 4. Discussion

Our analysis suggests that each hospital system will have to address cancellation problems at the level of each specialty and design interventions, addressing each particular medical provider, as was previously suggested but not demonstrated [1]. The novel finding of the study is a demonstration of significant variations between surgeons operating in the same service line for some specialties. This finding is a step further compared to prior observations [5,9,10,14,15,18,20,22,23,31,32,56]. Some surgical service lines presented homogenous provider cancellation frequencies, but that was not a uniform finding. This suggests that provider variability is the dominant driver for some service lines. Heterogeneity of the underlying reasons for case cancellation had been suggested before, but our study utilized a much larger sample [57,58]. The reasons for heterogeneity cannot be ascertained using the current study, suggesting that deployment of lean six sigma, guided interviews, or other exploratory techniques can more accurately address this problem [21,51].

The primary reasons for cancellations were date change (18.6%), patient cancellation (13.3%), and patient no-shows (10.9%). These findings are consistent with some of the prior literature, but cancellations are highly dependent on the specificity of the studied site [17,20,22,26,31,32,33,39]. Interestingly, medical-related problems (14.9%) were not our study′s top drivers for cancellations [8,39]. Instead, administrative reasons (28%) and increasing patient no-show ratios (10.9%) were more common and similar to prior studies [26,38,43,59]. The high variability of cancellations suggests that utilization of lean sigma six, or other techniques, has to be precisely applied to each surgeon and service line with high volume/high cancellation rates. [60]. Conversely, utilizing a system-wide solution may not effectively work, as was our assumption behind the presented project. This is a potential explanation as to why several solutions addressing operating room cancellations have not been successful in prior publications [25,41,47,48,50,52,53,59]. The solution to excessive cancellation needs to be targeted to the service line and, in some cases, specific healthcare providers. The majority of cancellations occurred before admission to the hospital (73.1%), underscoring that there is a window of opportunity to fill the emerging openings in the operating room schedule [21,29].

The highest cancellation rate seemed to be associated with Mondays (21.7%), Tuesdays (22.3%), January (10.4%), August (9.4%), and some national holidays. A prior study demonstrated that August and September had the highest number of cancellations [28]. However, time variation had a statistically insignificant impact on the cancellation rate compared to the service line and provider. This underscores the benefit of systematical analysis over observational studies [5,8,12,14]. This observation may be more applicable to hospitals not suffering from frequent shortages in staff or equipment [5,11,14,41].

Our data are consistent with some prior observations demonstrating that pre-surgery and patient-driven factors were the most common reasons for cancellations [10,13,24,25,26,27,33,34,35,36]. Using standardized analysis, several improvements could be suggested within the workflow. Since administrative and patient-driven reasons were dominant, addressing them may yield the most significant improvements. Administrative issues are of particular concern as they demonstrate system deficiency at the organizational level [4,6,10,24,25,26,27]. More efficient record-keeping should eliminate duplicate errors and scheduling errors. Addressing “date change” needs further exploration as the current data set is not sufficient regarding the nature of the cancellations. Several patient-related reasons (poor preparation, not fasting before surgery, lack of pre-operative labs) could also be addressed at an organizational level as well. Medical reasons were in the minority, but they may be very complex to address [3,6,10,27]. These pre-hospital cancellations should be treated as operational issues. The heterogeneity of the cancellations forces an approach from several different points of view to reduce the rate of cancellations. Some studies have recommended pre-surgical clinics, but our data suggest that its deployment would not address the majority of cancellations in our cases. Some of the proposed solutions are relatively easy to address, while others will need exploratory techniques (Supplemental Figure S5). The critical question is: what is the too-high versus the acceptable rate of cancellation? Economic pressure put on current U.S. hospitals may put undue pressure on OR operators, resulting in an increased risk of harm. A “zero cancellation” mantra is an axiomatic goal, but careful analysis of data should find the optimal balance when the cancellation rate is low enough without affecting risk/benefit ratio. Particular attention should be paid to cases related to cancellations after the patient reaches the operating room, particularly after induction [31]. They may signify near-misses, or misses, a potential sign of system underperformance similar to those seen in a situation where pressure is applied to perform cases even if a patient is not optimized. Some of the operators in our study had a very low rate of cancellation, but it is unclear if this below-average cancellation rate is related to an increased rate of adverse events.

Our study suggests several other exciting ideas for future investigations. The likelihood of cancellations was inversely proportional to the volume of the provider. This suggests higher volume does not increase cancellation rates and is consistent with prior findings [24]. It may suggest that operators with higher volumes are more efficient in utilizing the OR, but this correlation was relatively weak. Having a much larger study sample would allow for regression analysis to estimate the contribution of specialties, subspecialties, and operators to the cancellation rate. Conducting similar analyses in several other hospitals could answer the question of the generalizability of our findings. We would expect geographically limited generalizability, as several factors can affect performance in different geographical locations [4,7,10,11,12,13,18,20,25]. We demonstrated that a group of surgeons from different service lines disproportionally contributed to the overall cancellation rate, but this may be a local effect of their interaction with our hospital system. The top three services experiencing cancellations were GE, cardiac surgery, and orthopedics, compromising 60% of all cancellations. This was partially but not universally seen in other studies [8,10,17]. Again, this underscores the point that the underlying causes of cancellations have to be analyzed from the perspective of the individual service within the context of the particular healthcare system. However, our study does not allow for a more in-depth analysis of cancellation reasons.

Our data does have some limitations. The definitions provided by EPIC are too vague and broad. EMRs are subjected to bias [61]. Though they allowed for precise time-stamps of cancellations in the OR workflow, their ambiguity precludes further conclusions based on the provided data. The data demonstrated that an approach for cancellation needs to be focused on a particular group of operators within a given service line. Even though we had over 4000 cases, several more in-depth analyses were not possible considering data granularity. The whole system is managed by one administrative body, so the effect of different management styles cannot be ascertained. Notably, in other settings, the service line with the highest heterogeneity may differ [1,10,24]. However, identifying the operator and service line within the service line allows for the precise deployment of quality improvement projects, such as lean six-sigma, instead of deploying a broader approach, such as a preoperative clinic [2,38,46,48,50,56,60]. This is an interesting finding considering that several other studies suggested indirectly focusing on hospital- or service-level only. Focusing process improvement on the recognized “hot spot” allows for a precise focus of the effort, which is a considerable saving considering the size of some of the practice and related Q&I efforts to improve them. Though we believe that our study can be generalizable to the academic-located and urban hospitals in the U.S., a multicenter study would be necessary to find common motifs [6,25,26,41,54,56,60,62]. Even though the studied hospital characteristics are relatively common in the U.S., several specific differences will exist outside North America [4,8,10,13,15,17,23,32,35,62]. The collection of EMR records is inevitably susceptible to entry bias, as several cases had logs created in error or were duplicates, underscoring potential bias [63]. The study was conducted one year after the introduction of EMR; thus some staff may not be familiar with its use despite using a standardized educational approach [61,63]. A significant percentage of cases had no defined reason for the cancellation. Furthermore, very little insight can be gathered about the clinical nature leading to case cancellation. A review of records would be helpful, but it is a significant task as it would require the manual and retrospective abstraction of several cases [33,39,64].

The study′s strength is that our analysis was conducted in a highly regimented way. The methodology can be used as a template by which to compare different hospitals. Our surgical operators contributed to our practice for a long period. All surgery was conducted by one surgical center with few staff or protocol changes. High protocolization of the techniques increased data homogeneity. Some of our study′s observations have been corroborated by other observations [28]. Most of the cancellations were related to patient-related issues, but administrative reasons played a more frequent role [8].

## 5. Conclusions

Causes for cancellation should be reviewed at the level of providers within the service line with high inter-operator variability. Identifying service lines with low inter-operator variability suggests a more generalized solution to the cancellation problem.

## Figures and Tables

**Table 1:** Cancellation characteristics were defined depending on timing and were divided into clusters depending on if the cancellation occurred: before the patient reached the hospital, in the pre-operative area, or with the patient inside the operating room. Cancellations taking place before the patient reached the hospital were divided into patient-related, clinical, and administrative, with descriptors provided below. Cases were tallied by frequency and % of total cancellations. Yellow represents cancellations that were patient related; red represents cancellations that were clinical in nature; blue represents cancellations that were secondary to administration; orange represents cancellations that were miscellaneous and did not fit in the other groupings.

	Cluster	Reason	Additional Info	N; % Total			
**Cancelled before patient reaching hospital**	Patient-Related	Patient no show day of surgery		455 (10.9%)			
		Patient canceled		552 (13.3%)			
		No ride/weather/traffic		76 (1.8%)			
		The patient did not follow instructions	Did not take medications $Not adhering to pre-operative fasting$Poor bowel prep$No adequate pre-surgical workup	108 (2.6%)			
		Miscellanea	A patient seeking second opinion $Patient family refused $Patient positive for drugs/alcohol	27 (0.6%)			
	Clinical	Patient illness	Medical complication (diabetes, anticoagulation, blood pressure, other)$Cancelled for medical reasons$Abnormal tests	207 (5.0%)			
		Medical clearance and further med/surgical evaluation		163 (3.9%)			
		No longer indicated		203 (4.9%)			
		Misc (patient condition improved, patient expired)		46 (1.1%)			
	Administrative	Date change		773 (18.6%)			
		Scheduling error		134 (3.2%)			
		Canceled by administrator		111 (2.7%)			
		Miscellanea	Change of location$Faculty availability$Routine schedule rearrangement$Financial/authorization issues$The patient did not receive prep products or instructions$Others	146 (3.5%)			
	Other		Canceled for non-medical reasons$Surgeon misc. cancellation$Equipment not available	44 (1.1%)			
**Total number of cancellations before patients reach pre-op**					3045 (73.1%)		
**Total number of cancellations with patients in the pre-operative preparation area**					636 (15.3%)		
**Total number of cancellations with patients in the operating room**					68 (1.6%)		
**Total** **number of cancellations**						3749 (90%)	
**Duplicate record or log created in error**						416 (10%)	
**Total cancellations**							4165 (100%)

## Data Availability

Data is available upon reasonable request and subject to administrative approval.

## Supplementary Materials

The following supporting information can be downloaded at: https://www.mdpi.com/article/10.3390/healthcare11030357/s1, Figure S1: Workflow of the data analysis; Figure S2: Cancellation reason per top six service lines. Abbreviations: gastroenterology (GE), ophthalmology (Ophtho), interventional cardiology (Int. Card); Figure S3: Cancellation frequencies across six service lines. Abbreviations: gastroenterology (GE), interventional cardiology (Int. Card); Figure S4: Cancellation rate over time among top cancellation surgeon across different specialties; Figure S5: Possible solutions for common cancellations; Table S1: Cancellation reason per top six service lines.
