# Supplementary material for: An In-Depth Analysis of Providers and Services of Cancellation in Anesthesia Reveals a Complex Picture after Systemic Analysis
Source: Healthcare (Basel). 2023 Jan 26;11(3):357. doi: 10.3390/healthcare11030357 (PMC9914780; doi:10.3390/healthcare11030357)
Supplement: Supplementary file 1 [file healthcare-11-00357-s001.zip › healthcare-1898169-supplementary.pdf]

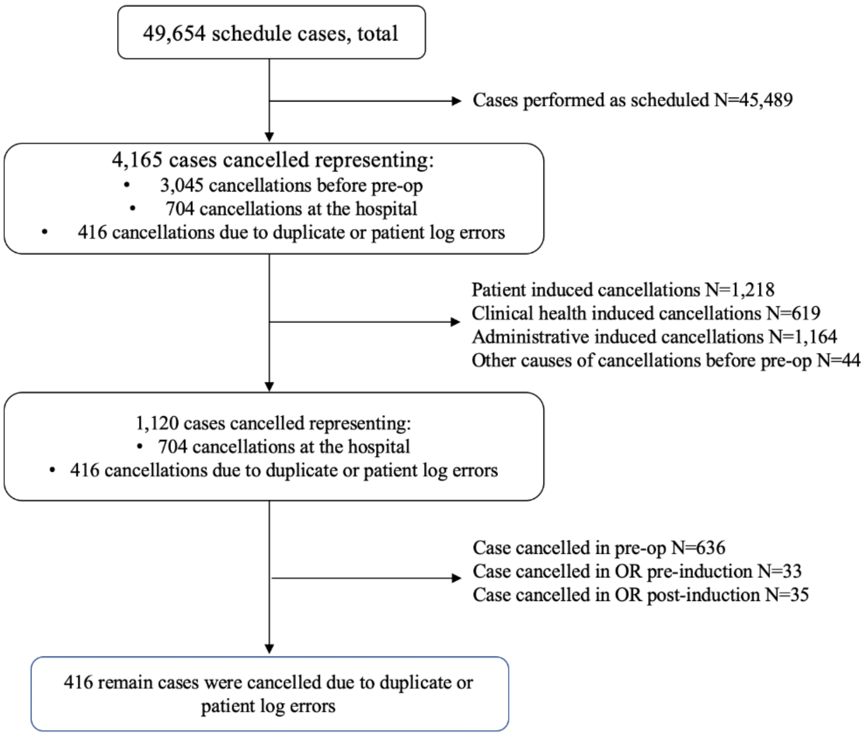

Figure S1. OR case breakdown in 2018.

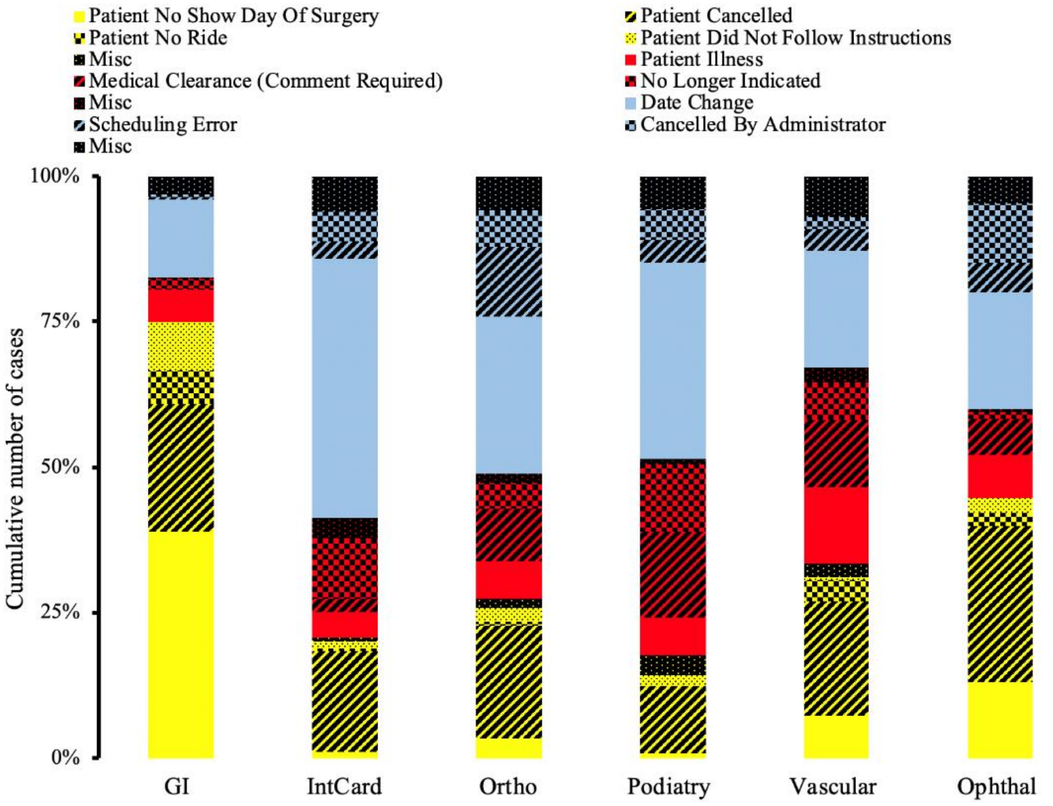

Figure S2. Cancellation reason per top six service lines.

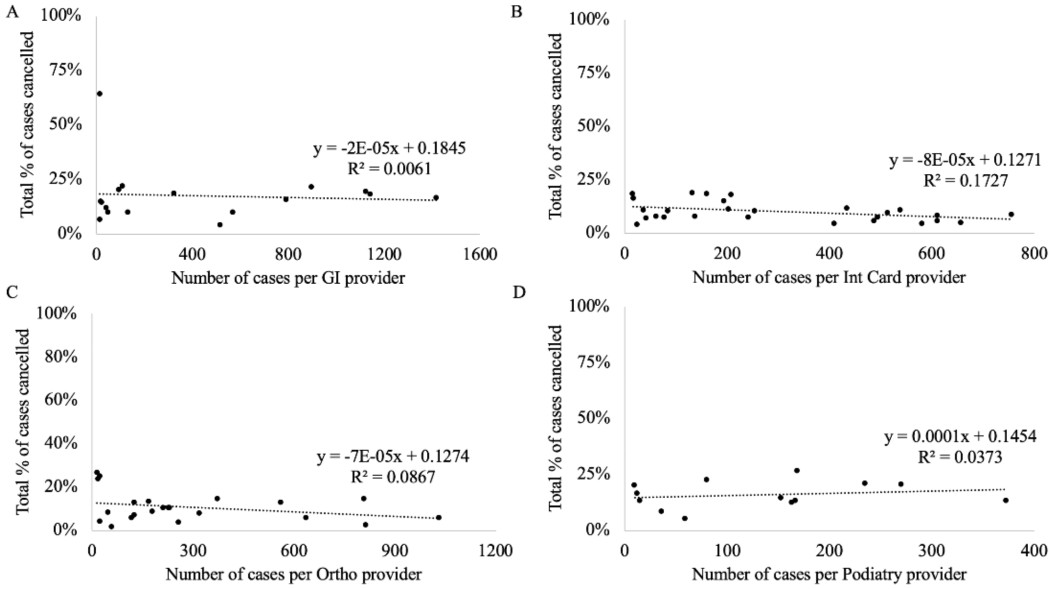

Figure S3. Cancellation frequencies across six service lines.

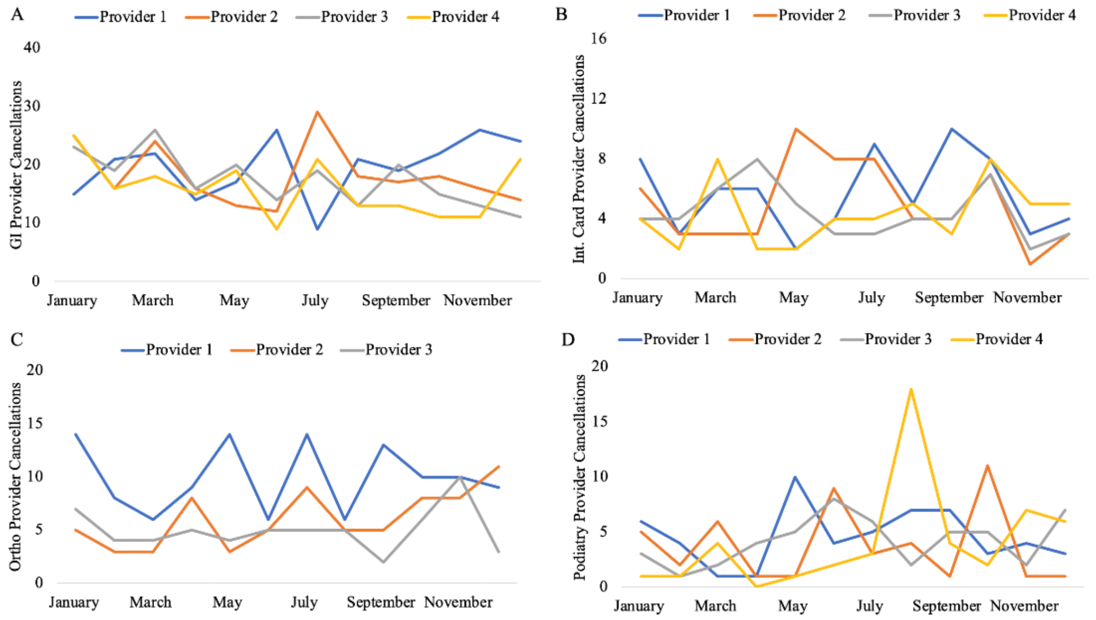

Figure S4. Cancellation rate over time among top cancellation surgeon across different specialties.

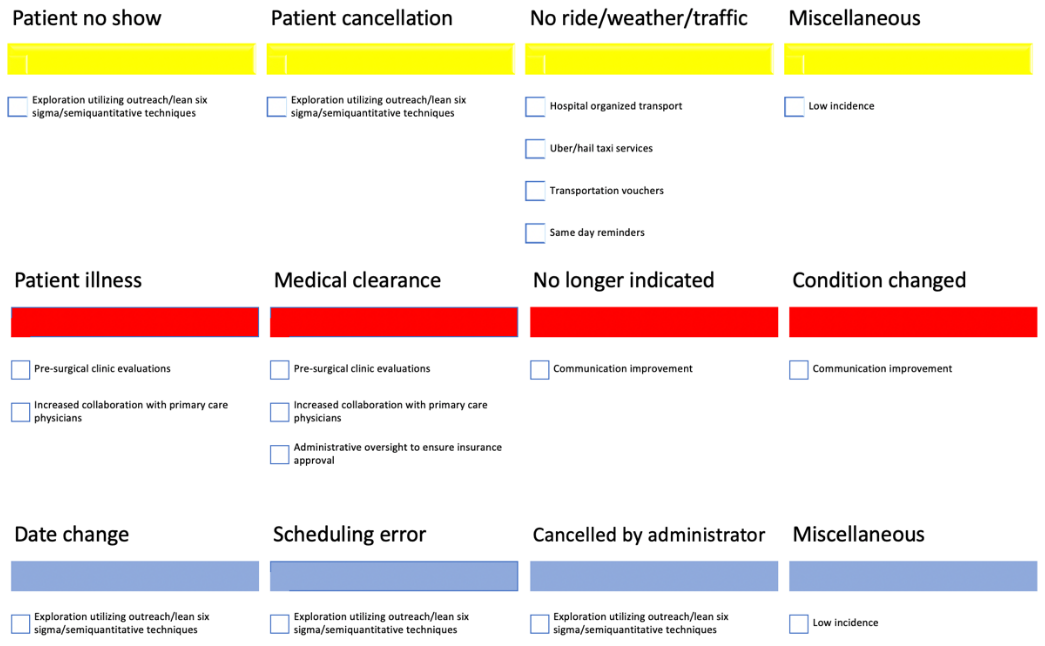

Figure S5. Possible solutions for common cancellations.

Table S1. Cancellation reason per top six service lines.

Clusters	GI	Int. Card	Ortho	Podiatry	Vascular	Ophthal
Patient no show day of surgery	381	4	13	2	14	25
Patient cancelled	213	68	77	24	37	51
Patient no ride	58	2	2	0	7	4
Patient did not follow instructions	80	5	9	4	1	5
Misc.	1	2	7	7	4	0
Patient Illness	54	18	25	14	25	14
Medical Clearance	1	9	36	31	22	12
No longer indicated	17	41	17	24	12	2
Misc.	2	13	6	2	5	1
Date change	131	175	106	71	38	38
Scheduling error	4	12	48	8	7	10
Cancelled by administrator	6	20	24	11	4	19
Misc.	29	24	23	12	13	9
Other	1	4	16	7	3	1
Cancelled in pre-op	119	231	80	39	48	40
Cancelled in OR pre-induction	1	8	8	3	1	6
Cancelled in OR post-induction	6	14	1	1	6	0
Log created in error	99	54	61	33	30	21
